# Electrical Impedance Changes at Different Phases of Cerebral Edema in Rats with Ischemic Brain Injury

**DOI:** 10.1155/2018/9765174

**Published:** 2018-06-04

**Authors:** Jiali Song, Rongqing Chen, Lin Yang, Ge Zhang, Weichen Li, Zhanqi Zhao, Canhua Xu, Xiuzhen Dong, Feng Fu

**Affiliations:** ^1^Department of Biomedical Engineering, Fourth Military Medical University, Xi'an, China; ^2^Equipment Division, People's Liberation Army 307 Hospital, Beijing, China; ^3^Department of Aerospace Medicine, Fourth Military Medical University, Xi'an, China; ^4^Institute of Technical Medicine, Furtwangen University, Germany

## Abstract

Cerebral edema contributes significantly to the morbidity and mortality associated with many common neurologic conditions. Clinically, a diagnostic tool that can be used to monitor cerebral edema in real-time and differentiate between different types of cerebral edema is urgently needed. Because there are differences in electrical impedance between normal cortical tissue and cerebral edema tissue, electrical impedance tomography (EIT) can potentially be used to detect cerebral edema. Accurate recording of the electrical impedance properties of cerebral edema tissue at different time points is important when detecting cerebral edema with EIT. In this study, a rat cerebral edema model was established; then, following the onset of ischemic brain injury, variation in the electrical impedance of cerebral edema was measured at different time points within a 24-hour period and the corresponding morphologic variation was analyzed. After the first six hours, following the onset of ischemic brain injury, the resistivity of brain tissue increased (p < 0.05); during this period, brain cell volume increased (p < 0.05) and the intercellular space decreased (p < 0.05) (behaving like cytotoxic cerebral edema). From 6 to 24 hours, the resistivity of brain tissue decreased; during this time, brain cell volume unchanged (p > 0.05) while intercellular space increased (p < 0.05) (behaving like vasogenic cerebral edema). These findings support the notion that EIT can be used to monitor the development of cerebral edema in real-time and differentiate between different types of brain edema.

## 1. Introduction

Cerebral edema is a frequent complication of several nervous system disorders including cerebral stroke, brain trauma, and other craniocerebral disorders. It is a life-threatening condition and puts the patient at risk for severe brain damage and death. According to Adnan et al., millions of people have died because of cerebral edema, and many patients suffer from all kinds of lifelong paralysis caused by brain edema, such as hemiplegia, and higher brain dysfunction [1]. One significant feature of cerebral edema is its rapid development, which has several different, distinct phases, such as the cytotoxic cerebral edema caused by the initial onset of ischemic brain injury and the vasogenic cerebral edema that occurs with the development of cerebral edema. In clinical practice, different types of cerebral edema require specialized treatment [2]. For example, the treatment of cytotoxic cerebral edema focuses on intracellular dehydration, while the treatment of vasogenic cerebral edema focuses on extracellular dehydration. Therefore, before treating cerebral edema, the different types of cerebral edema must be identified.

Existing brain imaging such as computed tomography and magnetic resonance imaging can be used to diagnose cerebral edema with significant space-occupying lesions, including brain midline displacement and ventricular deformation, but cannot differentiate the different types of cerebral edema [3]. Intracranial pressure (ICP) monitoring, another cerebral edema diagnostic technique, indirectly reflects the progression of craniocerebral injury by monitoring the changes in ICP. Although this technology can monitor cerebral edema in real-time, its invasive nature and cost make it not well accepted in clinical practice. Consequently, a noninvasive diagnostic tool to monitor cerebral edema in real-time and differentiate types of cerebral edema is urgently needed [4].

Electrical impedance tomography (EIT) is a safe, noninvasive, real-time, and functional imaging technique. EIT images the electrical impedance distribution of an organ by safely injecting electrical currents into human body and measuring the boundary voltages through surface electrodes [5]. Because differences in electrical impedance between normal cortical tissue and cerebral edema tissue exist, EIT can potentially be used to monitor cerebral edema and differentiate between different types of cerebral edema.

Accurate recording of the electrical impedance properties of cerebral edema tissue at different time points is an important element when detecting cerebral edema with EIT. Several studies have reported the electrical impedance of cerebral edema. According to Lingwood et al., in cerebral edema bioimpedance measurements have higher sensitivity than ICP monitoring [6]. In previous in vivo experiments, electrical impedance decreased in the injured brains of cats and rats compared to their normal controls [7]. Similarly, other studies showed that the impedance of cerebral hemorrhage was lower than that of the normal brain [8, 9]. The results of these studies showed that brain impedance is significantly different before and after the occurrence of brain injury; however, to the authors' knowledge, no study has investigated the electrical impedance changes of cerebral edema at different time points.

The aim of this study was to differentiate cerebral edema at different time points by measuring the electrical impedance of cerebral edema tissue. In this study, a cerebral edema model was established in rats by obstructing the right middle cerebral artery. Then, the electrical impedance of cerebral edema tissue at different time point was measured and the microscopic morphology of cerebral edema at different time points was analyzed quantitatively. Finally, the relationship between electrical impedance changes and microscopic morphology of brain edema tissue was discussed.

## 2. Materials and Methods

### 2.1. Experimental Setup and Data Analysis

The experiment was conducted strictly under the regulations of the Animal Ethics Committee of the Fourth Military Medical University, Xi'an, People's Republic of China. The Animal Ethics Committee approved the animal experimental setup.

**Figure 1:**
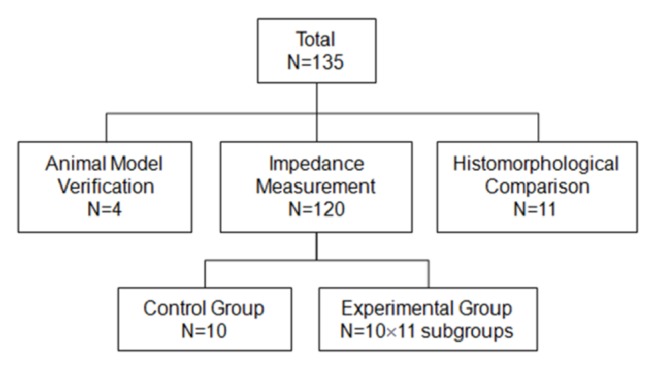
Rats were divided randomly into 12 experimental groups. N = number of rats in each group.

A total of 135 male rats (250–300g) obtained from Laboratory Animals Center of the Fourth Military Medical University were used for the animal model (n = 4), to measure electrical impedance (n = 120) and to compare histomorphological parameters (n = 11), respectively (Figure 1). To measure changes in electrical impedance at different time points of cerebral edema, 120 rats were randomly assigned to 12 groups: 1 control group and 11 experimental groups (representing the 1, 2, 3, 4, 6, 8, 10, 12, 16, 20, and 24-h time points, respectively). All rats were bred at a temperature of 23–25°C without a controlled diet.

### 2.2. Establishing the Ischemic Animal Model

**Figure 2:**
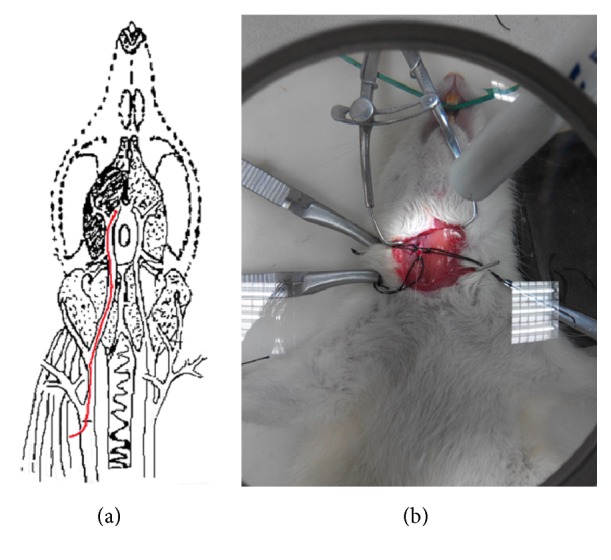
Preparation of the animal model. (a) MCA obstruction. (b) Actual photo of MCA obstruction. MCA, middle cerebral artery.

Each rat was anesthetized with an intraperitoneal injection of 10% chloral hydrate (3.5 mL/kg) and fixed in a supine position. A 1.5–2-cm neck incision was made 1 cm to the right of midline. The common (CCA), external (ECA), and internal (ICA) carotid arteries were separated along the sternocleidomastoid muscle. The proximal ends of the CCA and ECA were ligated. The proximal end of the ICA was tied with surgical suture. An incision was made approximately 1–2 mm proximal to the CCA bifurcation. The occluding suture was inserted into the ICA lumen. The suture was advanced along the ICA to the depth of 18 mm, thereby obstructing the right middle cerebral artery (MCA). The occluding suture was fastened with the suture tied around the proximal ICA and then the wound was stitched (Figure 2). In the control group, these steps were followed except for artery ligation and obstruction.

### 2.3. Validating the Ischemic Animal Model

The MCAs of four randomly selected rats were obstructed. The rats were euthanized after 2, 4, 10, and 24 hours of brain ischemia, respectively. Their brain tissue was harvested and placed in a fridge at a temperature of −20°C for 10 minutes. Brain tissue from the quadrigeminal cistern was cut into 1-mm slices along the sagittal line. The tissue slices were incubated at 37°C in 1% 2,3,5-triphenyltetrazolium chloride (TTC) buffered solution for 20–30 minutes. The stained brain tissue was scanned and photos were printed (HP Color Laser Jet Pro MFP M176; HP Inc., Palo Alto, CA, USA). Brian slices were analyzed using the Image-Pro Plus software (Version 4.5.0.19, Media Cybernetics Inc., Rockville, MD, USA,). The areas of the ischemic and nonischemic regions were first calculated, and then the volumes of the infarcted and noninfarcted brain tissues were obtained by multiplying the area by the slice thickness. Finally, the percentage of cerebral ischemia and the extent of cerebral edema were computed according to [10, 11]

(1) PIs=VL−VnVL×100%

(2) ECE=VLVR−1×100%

where *P*_*Is*_ is the percentage of cerebral ischemia, *V*_*L*_ represents the volume of the left brain, and *V*_*n*_ denotes the nonischemic volume of right brain, respectively.

### 2.4. Measuring System

**Figure 3:**
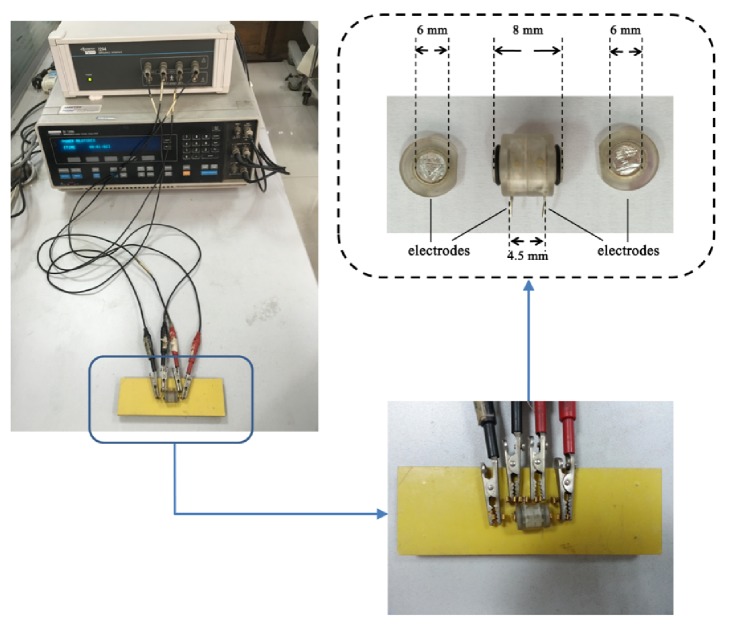
Impedance measurement system consisting of the Solartron 1260 impedance/gain-phase analyzer with a 1294A interface and the measurement box.

In our study, we established the impedance measurement system by employing a 1260 Impedance/Gain-Phase Analyzer (Solartron Analytical, Farnborough, UK) with a 1294A interface, controlled by the Zplot software (Scribner Associates Inc., Southern Pines, NC, USA), as shown in Figure 3. Additionally, the four-electrode method was adopted to measure the brain tissue impedance spectra because this strategy can significantly reduce the impact of electrode-tissue contact impedance [12]. Accordingly, a measurement box developed by our group was used to load the brain tissue for impedance measurement. The measurement box had a cylindrical cavity (6 mm in diameter and 8 mm in length) and four silver electrodes (Figure 1), where two disc electrodes with a diameter of 6 mm were located at both ends of the box and two ring electrodes with the same diameter were located in the middle. Each ring electrode had a rod to connect to the impedance analyzer and the distance between the two rods was 4.5 mm. The disc and ring electrodes served as exciting and measuring electrodes, respectively. In the experiment, after the removal of the brain, the brain tissue was loaded into the measurement box once it was trimmed. Before the impedance measurement, the measurement box was placed into a neonatal incubator (YP970; Ningbo Daiwei Medical Appliance Company, Ltd., Ningbo, People's Republic of China) to ensure a stable temperature (37±1°C) and humidity (90%). AC of 0.2 mA (r.m.s.) was used as exciting source while impedance spectra were collected from 10 Hz to 1 MHz in a logarithmic manner, with 10 points per decade. System stability was verified by measuring the impedance spectra of physiologic saline solution placed in the measurement box. (Theoretically, the electrical impedance of physiologic saline solution should remain the same at all frequencies if the measurement system is stable.)

### 2.5. Measuring Electrical Impedance

The rats in the 11 experimental groups underwent obstruction of the right MCA; they were euthanized 1, 2, 3, 4, 6, 8, 10, 12, 16, 20, and 24 hours, respectively, after obstruction. Then, the whole brain was removed immediately and the right brain was cut and loaded into the measuring box to measure electrical impedance. Before being loaded into the measuring box, the brain tissue was trimmed so that it fitted the space available in the measuring box. The rats in the control group were euthanized 24 hours after sham operation with an overdose of anesthetic and the impedance of the right brain was measured.

### 2.6. Histomorphological Comparison

The right MCAs of 11 rats were obstructed. The rats were humanely euthanized 1, 2, 3, 4, 6, 8, 10, 12, 16, 20, and 24 hours after operation. Brain tissue was perfused with 4% formaldehyde solution. Both left and right brain tissue was sampled and embedded in paraffin; 2–4-*μ*m thick slices were obtained and stained with hematoxylin and eosin. The slices were observed and analyzed under a light microscope with 200 × magnification. The Image-Pro Plus software was then used to extract the morphological parameters.

### 2.7. Data Analysis

The real (**Z**_**real**_) and imaginary (**Z**_**imag**_) parts of brain tissue impedance (**Z**) were obtained with the 1260 impedance analyzer. First, the modulus of electrical impedance was calculated:

(3) $$\begin{array}{c} \left|\;Z\;\right|=\sqrt{Z_{real}^{2}+Z_{imag}^{2}} \end{array}$$

where |**Z**| is the modulus of electrical impedance.

Then, the resistivity of brain tissue was calculated as follows:

(4) $$\begin{array}{c} \rho =\frac{\left|Z\right|\cdot S}{l} \end{array}$$

where *ρ* is the resistivity, **S** is the cross-sectional area, and **l** is the length of the measuring box.

To analyze changes in tissue resistivity caused by edema, the resistivity change rate was calculated:

(5) $$\begin{array}{c} k=\frac{\rho_{x}-\rho_{0}}{\rho_{0}}\cdot 100\% \end{array}$$

where **k** is the rate of change in resistivity, *ρ*_**x**_ is the resistivity of brain tissue **x** hours after ischemia, and *ρ*_0_ is the resistivity of normal brain tissue.

Data are presented as the mean ± standard deviation. Data were processed with SPSS version 14.0 (IBM Corporation, Armonk, NY, USA). One-way analysis of variance was used to analyze the data;* p *< 0.05 was deemed statistically significant.

## 3. Results

### 3.1. Measuring System Validation

Physiological saline electrical impedance measuring result is shown in Table 1. There is no significant difference between the electrical impedance of measured physiological saline at two different frequencies from 10 Hz to 1 MHz (p > 0.05). Consequently, the electrical impedance measuring system is stable enough to accomplish the measurement in this study.

### 3.2. Animal Model Validation

**Figure 4:**
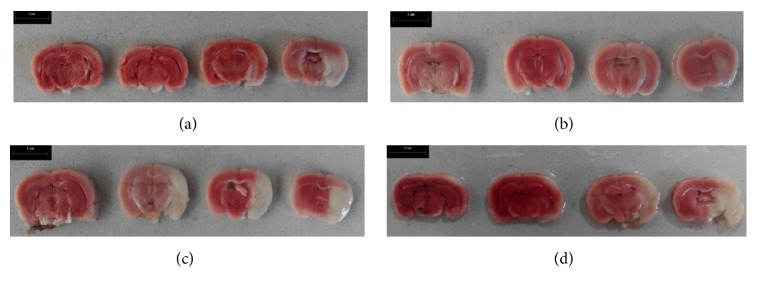
TTC staining of brain tissue 2, 4, 10, and 24 hours after the onset of ischemic brain injury, respectively. (a–d) Brain tissue from four different rats; each slice is 1-mm thick and the slices are arranged from the olfactory bulb to the pineal gland along the sagittal suture.

Figure 4 shows the results after TTC staining. Normal brain tissue appeared red while the ischemic brain tissue was white. The volumes of ischemic brain tissue 2, 4, 10, and 24 hours after ischemia were 45.55, 65.89, 20.80, and 49.57%, respectively, indicating that the ischemic model was successfully established. The degree of cerebral edema at four time points was 0.98, 4.98, 14.29, and 31.88%, respectively, suggesting that the extent of cerebral edema increases with time.

### 3.3. Electrical Impedance Measurement

**Figure 5:**
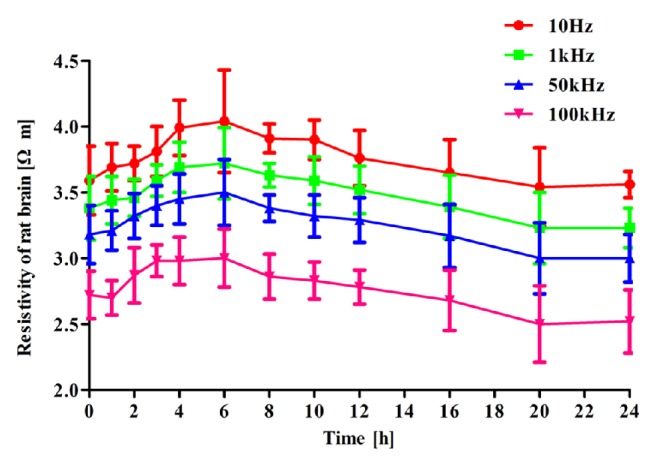
Changes in brain tissue resistivity at four different frequencies along the 24-hour time arc.

Figure 5 shows the changes in the brain tissue resistivity at four different frequencies and across the entire ischemia time range. Brain tissue resistivity decreased with increasing frequency (for example, at 8 hours, p < 0.05 at 10 Hz and 1 kHz, 10 Hz and 50 kHz, and 10 Hz and 100 kHz).

Within 6 hours after the onset of ischemic brain injury, brain tissue resistivity increased with time. For instance, at 10 Hz, brain tissue resistivity increased from the normal value of 3.59±0.26 Ω·m to a peak of 4.04±0.39 Ω·m (p = 0.001) after 6 hours of brain ischemia. Variation in brain tissue resistivity at the other frequencies showed the same trend (p = 0.003, p = 0.022, and p = 0.010 at 1, 50, and 100 kHz, respectively). Between 6 and 24 hours after the onset of ischemic brain injury, brain tissue resistivity decreased with time. At 10 Hz, brain tissue resistivity decreased from 4.04±0.39 Ω·m at 6 hours to 3.56±0.10 Ω·m at 24 hours after brain ischemia (p < 0.001). Change in resistivity at 1, 50, and 100 kHz was like the change at 10 Hz (p = 0.003, p = 0.022, and p = 0.010 at 1, 50, and 100 kHz).

### 3.4. Morphological Comparisons

**Figure 6:**
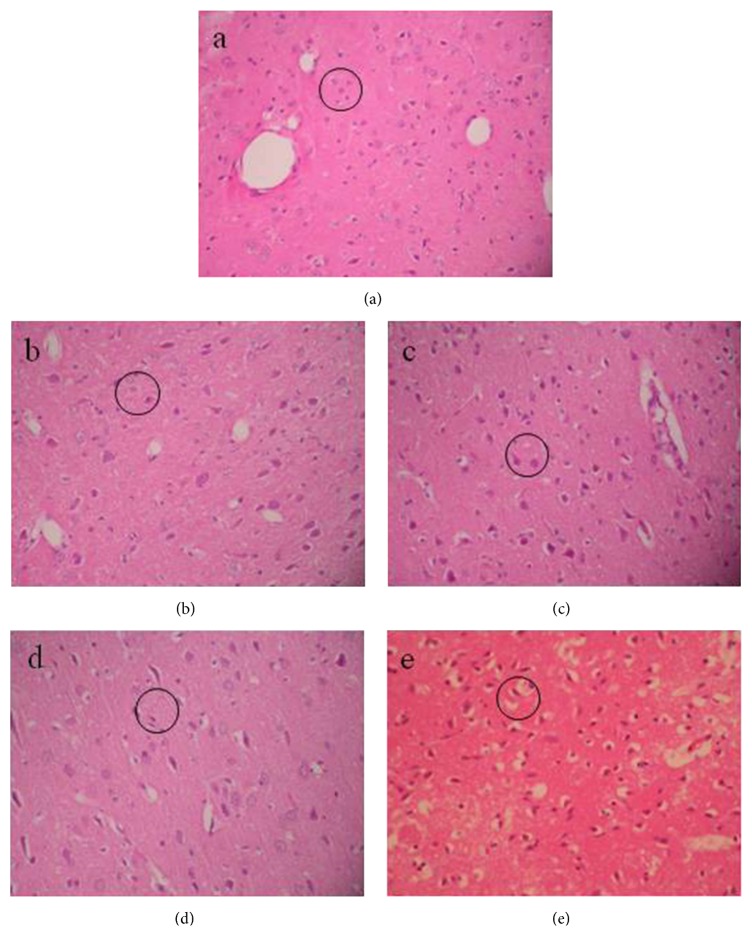
Microstructure of brain tissue under light microscope with 200 × magnification. (a–e) Microstructure of normal brain tissue, brain tissue at 2, 6, 10, and 24 hours after the onset of ischemic brain injury, respectively. The black circles denote the morphologic changes of brain tissue within the same area (the circles are in the same size).

The microstructure of brain tissue under a light microscope with 200 × magnification is shown in Figure 6. After 6 hours of MCA obstruction, the cell volume of the ischemic brain tissue (Figure 6(b)) was significantly greater than that of normal brain tissue (Figure 6(a)). Hypoxic-ischemic brain injury caused the neural cells to be more eosinophilic within 2 hours of MCA obstruction. Both factors indicated that brain tissue was at the early stages of cerebral edema. After 6 hours of MCA obstruction, the space between endothelial cells and the brain parenchyma had increased (Figure 6(c)), and chromatin condensation was also observed. After 10 hours, the vascular lumen had become occluded and the number of microglia had grown gradually (Figure 6(d)). Additionally, some neural cells had become necrotic, indicating that cerebral edema had become more severe. Twenty-four hours later, the brain tissue started to show signs of inflammation, encephalomalacia, and focal brain necrosis (Figure 6(e)), showing that brain tissue was at a stage of extremely severe cerebral edema.

**Figure 7:**
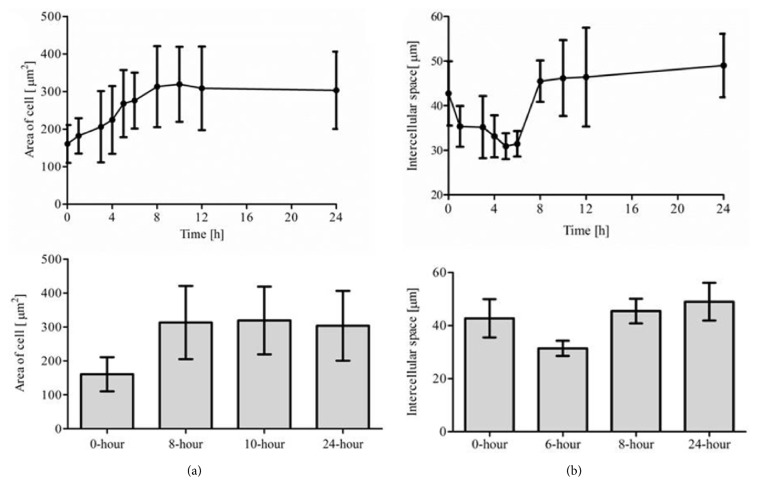
Changes in intercellular space and cell area with the time of ischemia. (a) Cell area. (b) Intercellular space.

Figure 7 shows the changes in intercellular space and cell volume with the time of ischemia. In this study, according to the approximation that both the cell volume and area are proportional to the cell size, we presumed that the cell volume grew with the increase of cell cross-sectional area, although the brain cells were not strictly circular. The cell volume gradually increased within 8 hours after the onset of onset ischemic brain injury (p < 0.01) and then merely remained constant from 8 hours to 24 hours (p > 0.05). The intercellular space first decreased within 6 hours after the onset of ischemic brain injury (p < 0.01 at 0 and 6 hours) and then significantly increased from 6 to 24 hours (p < 0.01).

## 4. Discussion

Unlike previous studies, this study comprehensively measured the change in resistivity of cerebral edema after the onset of ischemic brain injury at different time points along the 24-hour continuum and then quantitatively analyzed the corresponding microscopic changes in morphology.

In this study, brain tissue resistivity increased within the first six hours after the onset of ischemic brain injury. During this time, brain cell volume increased and intercellular space decreased (thus behaving like cytotoxic cerebral edema). Brain cell volume then decreased from 6 to 24 hours, whereas intercellular space increased (thus behaving like vasogenic cerebral edema).

**Figure 8:**
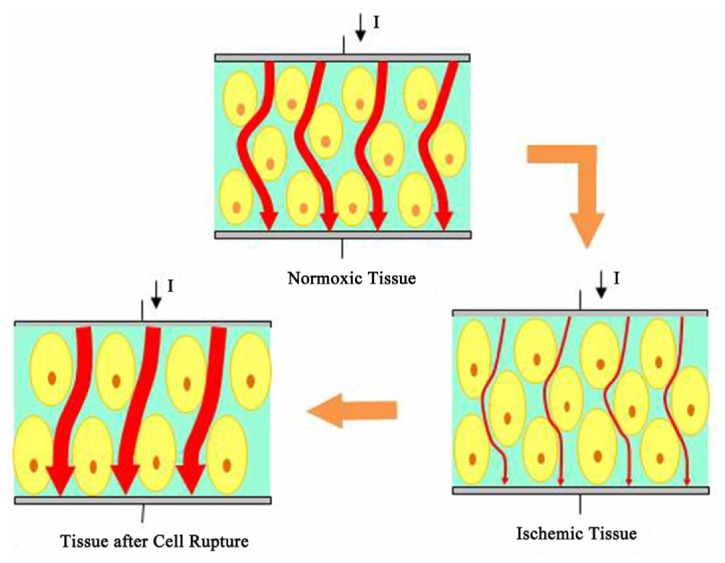
The current path through brain tissue changes at different phases of cerebral edema.

**Figure 9:**
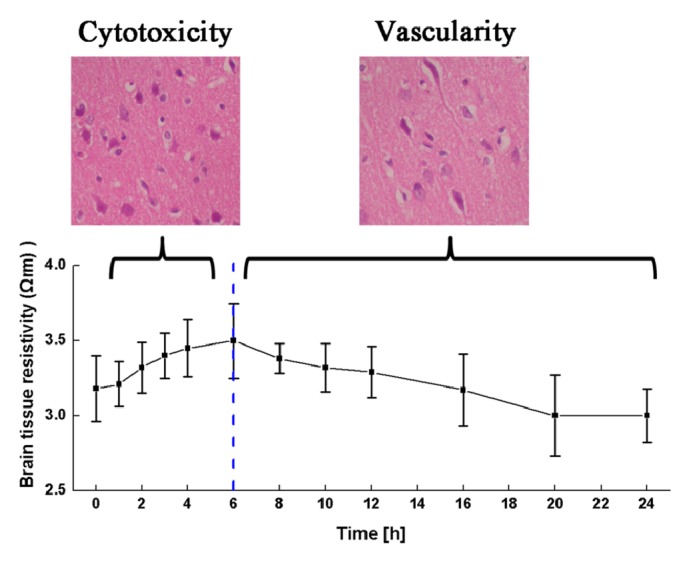
Brain resistivity changes of different types of cerebral edema at 10 Hz.

The change in resistivity could be related to the variation of brain cells and intercellular space. At an early stage of cerebral edema (approximately four hours after the onset of ischemic brain injury), brain cells gradually expand because of ischemia and hypoxia (Figure 8). Cellular expansion reduces the intercellular space; this decreases electrical current density through tissue under the same excitation (Figure 8), that is, tissue impedance increases (Figure 9) [13]. With prolonged ischemic time (approximately 4–8 hours after the onset of ischemic brain injury), both cell volume and intercellular space expand. Intercellular space expansion is mainly due to water entering the intercellular space through the blood vessels. At this stage, brain tissue resistivity increases if an increase in cell volume is dominant, whereas it would decrease if intercellular space expansion is prevailing [13]. Once ischemia further develops into severe brain damage, neurocytes become necrotic or rupture; this enhances cellular membrane permeability [14]. At this stage, the electrical current can easily pass through the brain tissue (Figure 8), which results in the further reduction of tissue impedance (Figure 8).

In this study, the increase in brain tissue resistivity lasted approximately for the first six hours after the onset of ischemic brain injury. This is in agreement with the findings reported by Lingwood et al. that brain resistivity increased six hours after anoxic injury [6]. In this study, brain tissue resistivity decreased between 6 and 24 hours after the onset of ischemic brain injury. Similar results were reported by Fujita et al. In their study, cold-induced cerebral edema in a cat model caused a 25% decrease in bioelectrical impedance of the lesion [7]. Dowrick et al. established an ischemic injury rat model using a methodology similar to the one used in this study. They reported that the brain resistivity increased within 30 minutes after the onset of ischemic brain injury, which is in agreement with the results of this study [8]. In general, good agreement can been seen between the results of this study and previously published reports.

### 4.1. Implications for Detecting Brain Edema with EIT

Since treatment of cytotoxic and the vasogenic cerebral edema is distinct in clinical practice, the most important purpose of detecting cerebral edema is to differentiate between these two types of cerebral edema. In this study, cell volume gradually increased within six hours after the onset of ischemic brain injury, a response that is typical of cytotoxic cerebral edema. During this time, brain tissue resistivity also gradually increased. Additionally, from 6 to 24 hours after the onset of ischemic brain injury, intercellular space started to increase, a response that is typical of vasogenic cerebral edema. During this time, brain tissue resistivity decreased. These phenomena suggest that a change in brain tissue resistivity can accurately reflect changes in tissue microstructure. Consequently, the characteristics of brain tissue resistivity can potentially be used to differentiate between different types of cerebral edema.

At present, ICP monitoring is the primary technique to monitor the development of cerebral edema in clinical practice. Because the cerebral edema changes the ICP, one can indirectly infer the degree of cerebral edema by measuring the ICP. However, at an early stage of cerebral edema, as it is the case with cytotoxic cerebral edema, the ICP does not change much even though brain cells have started to expand. Thus, ICP monitoring may not be a sensitive enough tool in the early stages of cerebral edema. In this study, after the onset of ischemic brain injury, brain tissue resistivity immediately increased, which suggests that monitoring brain resistivity may be more sensitive than monitoring ICP when detecting the development of cerebral edema, especially in the early stages. Therefore, EIT shows great potential as a tool to monitor cerebral edema. Additionally, compared with ICP monitoring, the noninvasive nature of EIT makes it more acceptable to clinical staff and patients.

### 4.2. Limitations of This Study

Although the total number of animals in the experiment was 135, the number in each treatment subgroup was limited. To examine variation in electrical impedance, the rats were dissected at different time points, which limited the number of rats at each time point. Further, changes in brain tissue electrical impedance during the development and progression of ischemic brain injury might be different between in vitro and in vivo models. Future in vivo studies should be designed to reduce the number of required subjects and to be more clinically relevant. Further investigations on other types of brain injury (such as hemorrhagic brain injury) could be undertaken, considering variation in electrical impedance and how patterns vary between different types of brain injury.

In this study, the electrical impedance and microscopic morphology of the cerebral edema tissue at different time points were measured. Because the changes of cell volume and intercellular space could be directly calculated from the morphologic results, we only analyzed the relationship between electrical impedance and intercellular space. In fact, tissue impedance is composed of three components: extracellular impedance, cell membrane impedance, and intracellular impedance. The condition of cell membrane will change as a result of the increase in cell volume after the onset of ischemic brain injury. Thus, we presume that the change in cell membrane condition might be another contributing factor to the variation in tissue impedance. In the future, in order to further quantify the relationship between tissue impedance and its three parts, the biological modeling method will be employed to model the measured tissue impedance to obtain the accurate impedance values of all components. Additionally, the ion concentration change of brain tissue also leads to the change of tissue impedance. Thus, the ion concentration change will be further investigated after accurate impedance of extracellular and intracellular space is obtained.

## 5. Conclusion

In this study, a rat animal model of cerebral edema was established. Then, the variation in electrical impedance of cerebral brain edema was comprehensively measured at different time points within 24 hours after the onset of ischemic brain injury. Finally, the corresponding variation in micromorphology was analyzed quantitatively. The results showed that brain resistivity rose at first and then decreased after ischemic brain injury (at all frequencies; p < 0.05). Resistivity peaked around six hours after ischemic brain injury (10–15% higher than before the onset of ischemic brain injury). Brain intercellular space decreased within the first four hours after ischemic injury and then increased, while cell volume increased within the first eight hours and decreased thereafter (p < 0.05). In conclusion, this research illustrated the changes in resistivity within the first 24 hours after ischemic brain injury and outlined the relationship between changes in electrical impedance and the micromorphology of brain edema tissue. These findings validate the feasibility of using EIT to monitor brain edema in real-time and differentiate between different types of brain edema and provide data that can be used to support further research in this field.

## Figures and Tables

**Table 1:** Electrical impedance and resistivity of physiological saline from 10 Hz to 1 MHz.

Frequency(Hz)	Electrical Impedance(Ω)	Resistivity(Ω·m)
1×10	1173.30±10.07	2.58±0.11
1×10^2^	1173.50±11.00	2.58±0.09
1×10^3^	1173.30±9.27	2.58±0.08
1×10^4^	1167.21±12.24	2.57±0.11
1×10^5^	1183.91±11.01	2.60±0.12
1×10^6^	1203.82±20.12	2.87±0.20
